# The Effects of Parental Absence on Children Development: Evidence from Left-Behind Children in China

**DOI:** 10.3390/ijerph17186770

**Published:** 2020-09-17

**Authors:** Mingzhi Mao, Lijun Zang, Haifeng Zhang

**Affiliations:** 1 School of Public Affairs, Zhejiang University, Hangzhou 310058, China; vera1991@zju.edu.cn (M.M.); lijunzang_zju@zju.edu.cn (L.Z.); 2 School of Economics and Center of Social Welfare and Governance, Zhejiang University, Hangzhou 310058, China

**Keywords:** parental absence, cognitive ability, test score, left-behind children

## Abstract

Parental care in early childhood is viewed as one of the most important factors that help foster children’s abilities. Using two nationally representative datasets collected in China, this paper examines the effects of parental absence on the short-term in-school outcomes and long-term educational achievement of left-behind children. The results show that parental absence is negatively associated with the development of left-behind children. Left-behind children have a lower cognitive test score and academic test score, and they are also less likely to attend a college. In particular, a mother’s absence seems to have persistent negative effects on children’s development. Mechanism analyses show that parental absence may result in a less healthy mental status of children and reduce children’s efforts in class. However, we do not find significant evidence that the exposure to left-behind children in class lowers the in-school outcomes of children.

## 1. Introduction

Parental care in early childhood is viewed as one of the most important factors that help foster the cognitive and non-cognitive abilities of children [1]. A vast literature has shown that the absence of parental care in early childhood negatively affect children’s development in health status, daily behaviors, and in-school performance [2,3,4,5,6,7]. A longitudinal study in Thailand finds negative impacts of parental absence on the school enrollment of left-behind children (LBCs), and shows the long-term absence of the mother appears to reduce the educational chances of LBCs [8]. A study examines the data from the Mexican household survey, and find higher emotional and behavioral problems among LBCs than non-LBCs [9]. Concurrently, a study in the Philippines shows the opposite results, that is, LBCs have better well-being outcomes than non-LBCs [10]. A systematic literature reviews the effects of international labor migration on mental health and well-being, and shows that the effects are not always negative but depend on the characteristics of LBC as well as other family characteristics [11]. Compared to international migration, a study uses a nationally representative panel dataset in China and shows that internal parental migration significantly increases the depression scores of children aged 10–11 [7].

Since the reform and opening up, China has witnessed a large-scale internal migration from less developed areas to developed areas. Meanwhile, migrant workers usually cannot bring their children to the cities due to the restrictions of the urban educational system [12]. As a result, a number of children become “left-behind children” who typically do not live with their parents for a long time and lack sufficient parental care. At the end of 2015, the number of left-behind children (aged 0–17) due to parental migration reached approximately 69 million (“Left-behind children” here refer to those “who live in their original domicile, but have not lived with their parents for over six months, as either one or both parents have migrated”), which accounts for 25.4% of the total child population in China [13].

A growing number of studies have examined the effect of parental absence on the human capital development of left-behind children, but the evidence is still far from a consensus [14,15,16,17,18]. For example, Li finds that parental migration leads to significant declines in left-behind children’s educational performance [14]. A study further distinguishes the impacts of the absence of one versus both parents [15]. Their findings show significant adverse effects of being left behind by both parents on children’s cognitive development, but much smaller insignificant impacts of being left behind by one parent [15]. A study estimates the effect of parental migration on students’ school behavior, and finds that parental migration negatively affects school behavior of left behind older students significantly, especially the class integration and personal behavior control [19]. One exception found no significant impact of parental migration on the math achievement of left behind children [20]. Furthermore, a study shows that parental absence has a significant negative impact on students’ long-term educational development [17].

In this paper, we employ the data from China Education Panel Survey (CEPS), a nationally representative middle school-based survey, to examine whether and how parental absence affects the educational development of middle school students in China. We contribute to this literature in four aspects. First, we look at the effects of parental absence on rural left behind children as well as their urban counterparts. The number of left behind children in urban areas increased from 14.7 million in 2005 to 28.3 million in 2015 [13]. However, less attention is devoted to the effects of parental absence on left behind children in urban areas. Second, while previous studies on left behind children extensively look at the short-term effects of parental absence, we also investigate the potential persistent effects of parental absence. Third, we uncover the potential mechanisms by using mental health as a mediator variable affecting students’ educational development. We also explored other mechanisms through which parental absence affects student achievement in middle school. Lastly, we attempt to examine the external effect of left behind children in a class on the outcomes of their classmates.

The CEPS data provide detailed demographic and family characteristics, which help us identify the family structure on whether the student lives with one or both of his/her parents, regardless of whether the student has a rural or urban Hukou (The Hukou system refers to the household registration system, which classifies people into two types, i.e., rural residents and urban residents. More details are explained in Section 2). We limited our sample to schools that randomly assign students into classes since the CEPS contains information on a school assignment rule, which helps eliminate the potential self-selection problem. Furthermore, we employ an instrumental variable strategy to address the endogeneity issues. Thus, this study estimates the causal effects of parental absence on the educational development of students in rural and urban areas of China. The CEPS also enables us to examine whether and how the Share of LBCs affects students’ academic achievements.

In this study, we show that parental absence has significant adverse impacts on left behind children educational outcomes. We find that left behind children have a lower cognitive test score and academic test score, and they are also less likely to attend a college. Our results are robust to a series of sensitivity tests. The effects of parental absence vary across gender, parents’ education level, and Hukou type, with more significant effects for girls, students with low-educated parents, and urban students. Further mechanism analyses suggest that parental absence reduces students’ mental health by increasing the likelihood of being depressed or unhappy. Students with parental absence tend to have a lower self-assessment on their behavior and campus life, such as more likely to be late for school, less likely to receive praise from the head teacher, and problems with class integration and social interaction with others. Our estimations also find no significant evidence in which the exposure to left behind children in class lowers the in-school outcomes of children.

We further examine the long-term effect of parental absence on students’ educational development using alternative data from the China Family Panel Studies. The results show that a mother’s absence is significantly and negatively associated with children development in comparison to a father’s absence. One percentage increase in the length of mother’s absence reduces the probability of graduating from senior high school and entering college by 6.7% and 6.5%, respectively.

The rest of the paper proceeds as follows. Section 2 provides the institutional background. Section 3 introduces the data and variables used in the empirical analysis. Our estimation strategy is discussed in Section 4. Section 5 presents the empirical results. Section 6 concludes the paper.

## 2. Institutional Background

Left behind children in China have attracted increasing attention, and there are many concerns that children are separated from their parents. China has experienced rapid economic development and unprecedented urbanization since the reform and opening-up policy, which have attracted people to move to large urban centers with employment opportunities. However, migrant workers usually cannot move their families to the cities due to the institutional segregation between rural and urban areas as well as because of their own financial constraints. For example, parents’ economic resources cannot support living with children in a destination city where they search for better employment opportunities. Therefore, parents have to leave the children in their hometown and ask relatives to take care of the children.

Except for financial constraints, the fact is that internal migration in China has always been controlled by the government, mainly through the household registration system, which is also known as the Hukou system. In the 1950s, the Chinese central government established the Household Registration System that registered all Chinese citizens as residents in a specific region, which is usually their birthplace and cannot easily be changed. Notably, the registration system classified people into two types—agricultural (rural residents) and non-agricultural (urban residents) [21]. Depending on the different types of registration, people’s access to public services differed from each other. At the same time, individuals’ “local” residency was also associated with eligibility for benefits provided by local governments, such as public education and social security. That is, people who have registered in one place could rarely enjoy benefits elsewhere [21].

China’s household registration system is often criticized for hindering the free movement of labor and restricting access to government-sponsored benefits. Migrants who move to some developed areas for employment rarely have access to the destination’s Hukou on which the social welfare system is based. Thus, they obtain fewer resources for their children’s development when compared to the natives. Because of a lack of urban household registration status (hukou), migrant children do not enjoy the same educational opportunities and resources as local children. For example, a study found that the majority of rural migrants in cities who do not possess Hukou are unable to enroll in public schools to receive free compulsory education [22] (In Chinese cities, two criteria are essential for public schools to admit students. First, students must reside within the local school district in the city, and, second, students must be registered in the school district as well, i.e., having local Hukou [22]).

Since 2012, the Hukou system has undergone a variety of reforms and received increasing attention, as the Chinese government started to propose a unified Hukou system to break the dual division pattern in realizing urbanization. Recent Hukou reforms aim to integrate migrant children into the local public education system. Even though the official policy is clear that migrant children should not be charged extra fees for public school education, there are still other barriers for the migrant parents. For example, they are required to provide a continuous record of social insurance contributions and other documents so that their children can have access to local public education. Even though migrant children can receive nine-year compulsory education in the inflow areas, they may still not be able to attend the college entrance examination. As a result of the cost or uncertainty of educational opportunities in inflow areas, the migrant parents may choose to leave their children in their hometown.

## 3. Data and Variables

The primary data used in the paper are from the China Education Panel Survey (CEPS), which is conducted by the National Survey Research Center at Renmin University of China (The baseline survey of CEPS was completed in the 2013–2014 academic year, conducted by the National Survey Research Center (NSRC) at the Renmin University of China. The CEPS applies a stratified, multistage sampling design with probability proportional to size (PPS) by randomly selecting a school-based, nationally representative sample across mainland China). The CEPS is a large-scale, nationally representative, longitudinal survey starting with two cohorts—the 7th and 9th graders in the 2013–2014 academic year. The baseline sample consists of approximately 20,000 students in 438 classrooms of 112 schools in 28 county-level units in mainland China.

Although the Chinese education system requires the random assignment of middle school students to classes, some schools may not strictly comply with the rule in practice (As documented in *Article 22 of the Compulsory Education Law of the People’s Republic of China*, “People’s governments at or above the county level and the administrative departments for education shall promote balanced development among schools by narrowing the differences in the conditions for school running, and they shall not divide the schools into key and non-key schools. Furthermore, the schools shall not divide the classes into key and non-key classes.” *Article 57* of the Law further regulates that “a school shall be ordered to make rectification within a time limit by the administrative department for the education of the people’s government at the county level” and “if the circumstances are serious, the person directly in charge and the other persons directly responsible shall be punished according to law” if *Article 22* is violated). We use the following steps to identify the schools that may not strictly comply with the random assignment rules. First, each principal is asked to answer whether students are randomly or evenly assigned to classes when they are enrolled in school and whether students are transferred to another class during the subsequent middle school period. We drop the schools that reported a nonrandom assignment during entrance or a redistribution in grades 8 and 9. Second, head teachers are requested to report whether students in the school are assigned by test scores. We drop the schools that may assign students by test scores. As a result, the final sample of CEPS consists of 10,532 students distributed over 256 classrooms and 64 middle schools.

The CEPS contains rich information on each student’s performance, which enables us to have multiple measures for students’ academic achievements, namely, cognitive test scores, academic test scores, and educational aspiration. It is well documented that cognitive test scores and academic test scores play important roles in determining the enrollments of high school. The CEPS conducts standardized cognitive ability tests for students in each grade, respectively, and collects mid-term examinations provided by the school administration offices (The school administration offices are requested to provide students’ academic test scores in the core subjects (Chinese, math, and English) in the mid-term examinations. These subjects (Chinese, math, and English) are compulsory and are the main components in the standard tests for admission to senior high school. Test scores in the core subjects are there for a consistent measure of academic achievement across students from the same grade in the same school). To measure educational aspiration, the CEPS asked each student the following question: What degree do you want to attain? The answers include “dropping out of school right now,” “middle school,” “technical secondary school,” “vocational high school,” “senior high school,” “3-year college,” “4-year college/bachelor’s degree,” “master’s degree,” and “doctoral degree.” We construct a dummy variable that equals 1 if the desired educational attainment is at least college.

We are interested in whether being a left behind child would affect his or her own academic performance. Exposure to left behind children in class would have an adverse effect on children’s academic performance. Hence, we construct two key independent variables. One is parental absence defined as a dummy variable equal to 1 if the student is a left behind child. The other is share of LBCs defined as the share of classmates who are left behind children.

We also control some predetermined individual variables (such as age, gender, Hukou, ethnicity, mother’s education, and father’s education) and class-level variables (such as class size and share of better-educated parents in class) that may affect children’s educational development [7,15,23,24]. Definitions of these variables are presented in Table 1.

The CEPS data also cover rich information about students’ self-assessment on mental health status, behaviors, and campus life as well as the interaction between student-student and student-teacher, which enables us to explore further the black box inside the effects of parent-child separation. We defined a set of potential channel variables (as listed in Table 1) according to children’s responses to the questions.

Table 1 summarizes the statistics of the variables. On average, 19.8% of students in the sample are left behind children. However, the standard deviation (0.398) of Share of LBCs indicates inequality in peer composition across classes. Table 2 presents the summary statistics of the outcome variables for LBCs and non-LBCs students. It can be seen that non-LBCs students have better academic performance than LBCs students.

## 4. Methodology

We implement the following linear-in-means model to estimate the effects of a parental absence.

(1) $$Y_{ics}=\beta_{0}+\beta_{1}*Parental\;absence_{ics}+\beta_{2}*Share\;of\;LBCs_{-ics}+\phi X_{ics}+\varpi_{g}+\lambda_{s}+\delta_{gs}+\varepsilon_{ics}$$

where *i* denotes students, *s* denotes middle schools, and *c* denotes classes. $Y_{ics}$ is the outcome measure for student *i* in class *c* of school *s*. $Parental\;absence_{ics}$ is a dummy variable denoting whether student *i* is/was a left-behind child who is/was absent of parental care. $Share\;of\;LBCs_{-ics}$ denotes the share of classmates who are/were absent of parental care in student *i*’s class excluding student *i*. $\lambda_{s}$ is a school fixed effect that controls for the potential endogenous sorting of students across schools based on unobserved factors. The term $\varpi_{g}$ is a grade fixed effect that controls any unobserved grade-specific shocks common to all schools. Furthermore, we include school-specific grade trend effects $\delta_{gs}$ to account for any unobserved school-specific grade-varying factors. The covariate vector $X_{ics}$ contain individual and class characteristics that are essential determinants of students, including students’ age, ethnicity, sibling size, gender, Hukou type, parents’ education levels, class size, and share of better-educated parents in class. Lastly, $\varepsilon_{ics}$ is the error term. Throughout the analysis, we cluster the standard errors at the class level.

The coefficients $\beta_{1}$ and $\beta_{2}$ are parameters of interest. $\beta_{1}$ captures the effects of parental absence on our own educational outcomes of left behind children, while $\beta_{2}$ captures the external effects of exposure to left behind children in class on the educational outcomes of students in the class. An ordinary least squares (OLS) estimation of Equation (1) may obtain biased estimates of $\beta_{1}$ and $\beta_{2}$ because both being a left behind child and share of LBCs may be endogenous. To overcome the potential bias, we employ an instrumental variables strategy to address the possible endogeneity of being a left behind child. We use Stata 16.0 to conduct the regression analysis based on Equation (1) and the Stata program codes are available upon request.

Furthermore, we follow the literature to exploit the mandatory rule that requires schools to randomly assign students to classes in school to address the possible class sorting [25,26,27,28,29]. While some schools might declare that they follow the randomized rules, but did not, we cannot identify such schools and eliminate them from the sample.

**Figure 1:**
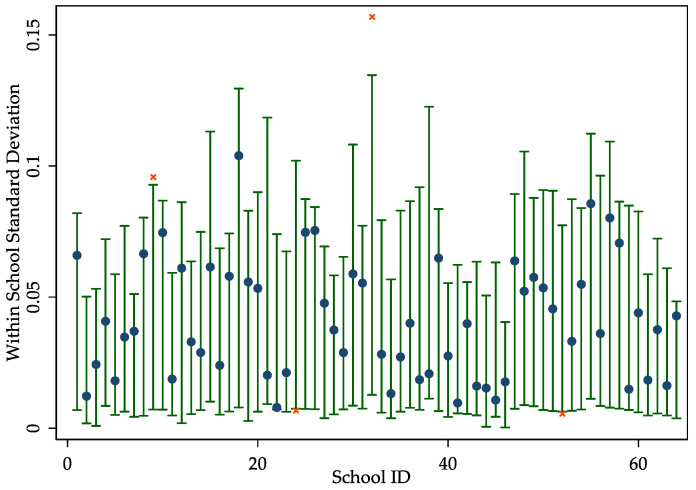
Monte Carlo Simulations of left behind children (LBCs) share. Notes: The figures present the Monte Carlo simulations for the within-school standard deviation in the share of LBCs. Vertical bars represent simulated 95% confidence intervals for within-school standard deviations in the share of LBCs. Scatter points represent actual within-school standard deviations for each school. Filled circles indicate that the actual standard deviation is within the simulated 95% confidence interval, whereas x’s indicate schools with standard deviations outside the simulated confidence interval.

To test whether the rule works well, we follow the approach of Lavy and Schlosser to perform a Monte Carlo Simulation [30]. Specifically, for each student *i* in the sample, we randomly generate the parental absence status of *i* using a binomial distribution function with *p* equal to the average proportion of students with a parental absence in the school across four classes. We then compute the within-school standard deviation, using the residuals from regressions of LBCs share on school fixed effects, grade fixed effects, and school-specific grade trend effects. We repeat this process 1000 times to obtain an empirical 95% confidence interval of within-school standard deviations. Figure 1 summarizes the simulation results for students with a parental absence. We find that the observed standard deviation in the LBCs share is within the 95% empirical confidence interval for 90% of schools, which is consistent with a random process.

We also investigate the validity of the identification strategy by looking at whether the variation in the share of LBCs within schools and grades is related to the variation in several predetermined student characteristics. If this assignment process is truly random, the students should be similar across these predetermined characteristics [29]. We run separate regressions in which the dependent variable is students’ predetermined characteristics, and the share of LBCs in the class is the independent variable by adding grade and school fixed effects and school-specific grade trend effects. The evidence in Table 3 supports the validity of the above identification assumption. That is, the students’ predetermined characteristics are balanced across classes with a different share of LBCs students.

These statistical tests suggest that variation in the share of LBCs is uncorrelated with observable and unobservable changes within schools. A final potential threat to our identification strategy is if students strategically transferred to another school or class. While we cannot directly test for this with our data, we find it highly unlikely in the context of the Chinese middle school education system, where students cannot generally transfer from one public school to another unless they relocate to another city province (In the Chinese educational system, middle school students are assigned to the classroom at the beginning of the 7th grade and take the same courses throughout three years of middle school. Students are required to take three core subjects-Chinese, math, and English and a set of subsidiary subjects. During a regular school day, students remain in the same classrooms all day, and different teachers come to the classroom to deliver subject-specific letters).

## 5. Results

### 5.1. Ordinary Least Squares Estimation

Table 4 reports the estimation results for Equation (1). We report the results without controlling for individual controls in the odd columns, and those with individual controls in the even columns. We first examine the effects of parental absence on students’ educational outcomes, as measured by a cognitive test score, an average academic test score, and educational aspiration. The coefficient of parental absence is negative and statistically significant, which suggests that the absence of parental care may have an adverse effect on the academic outcomes of left-behind children. The effect is not small. As shown in Columns (2) and (4), on average, a parental absence tends to decrease a cognitive test score and an average academic test score of left behind children by 0.167 and 2.612 points, respectively. Columns (6) indicates that parental absence will lead to a 2.2 percentage point decrease in the likelihood of attending the college.

However, we find the coefficient of the share of LBCs is statistically insignificant, which suggests no evidence is supportive of the external effect of exposure to LBCs students in a class.

### 5.2. Instrumental Variable Estimation

As noted earlier, the status of being a left behind child may be endogenous due to some unobservable factors. To address this potential endogeneity, we conduct the instrumental variable estimation. An instrumental variable should be correlated with the status of being a left behind child, but does not directly affect children’s educational performance. Since parental absence at the school level reflects the overall parenting behavior of a school district, the parenting behavior in other classes in the same school are likely to affect the parents in a particular class but will not affect the students’ educational performance. Therefore, we use the share of LBCs within the same school as the instrumental variable.

As shown in Table 5, the F statistics of the first stage indicate that the weak instrument problem may not be a significant issue. Columns (1)–(3) in Table 5 presents the results of IV estimations. The results are similar to the baseline estimations, that is, parental absence has adverse effects on students’ educational outcomes.

### 5.3. Robustness Tests

We further conduct a series of robustness tests to check the baseline results. We first examine whether the effects of parental absence vary across subjects (i.e., Chinese, Math, and English). We both conduct an ordinary least squares (OLS) estimation and an instrumental variable (IV) estimation. The results in Table 6 suggest that the signs and magnitudes of the effects for each subject are similar to those reported in Column (4) of Table 4 and Column (2) of Table 5.

**Figure 2:**
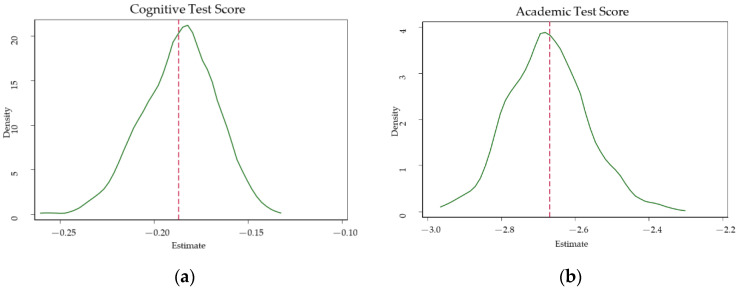

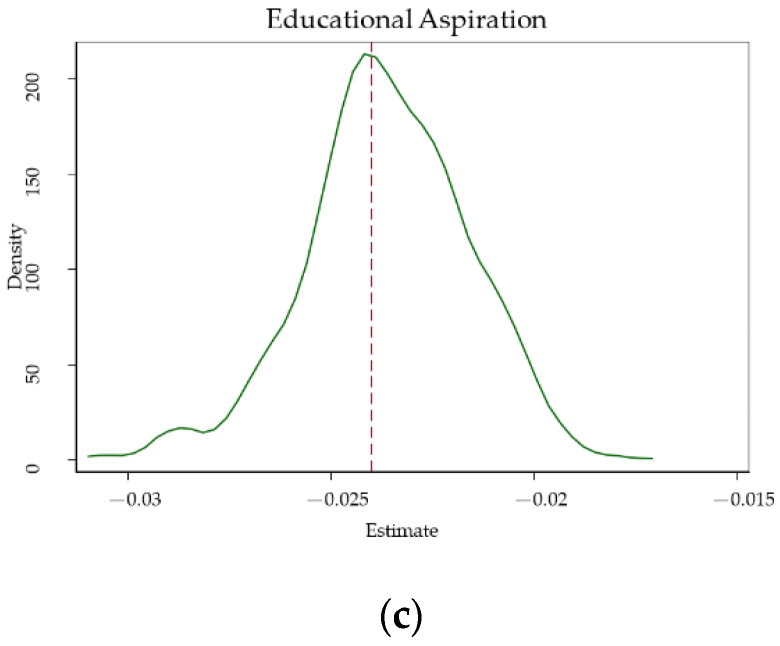
Distribution of estimated coefficients on student outcomes. Note: The figures present the distribution of estimated coefficients of parental absence on student outcomes, (**a**) distribution of estimates on cognitive test score, (**b**) distribution of estimates on the academic test score, and (**c**) distribution of estimates on educational aspiration.

We also follow the approach to conduct a placebo test. Specifically, we check whether the regression results change dramatically if we randomly drop several schools from the baseline sample [31]. To maintain a sufficient sample size, each time we randomly drop two schools from 64 schools, and repeat it 1000 times. Figure 2 shows the probability density distribution of the regression coefficient $\beta_{1}$. The distributions are strictly around their respective baseline estimates. Overall, it shows that our estimations are unlikely to be biased due to containing non-randomly assigned schools.

### 5.4. Heterogeneity

We then explore the potential effect of heterogeneity among students. Again, IV estimation is used for the estimations. We first consider whether the effects vary across gender, as shown in Panel A of Table 7. For boys, the cognitive tests score and academic test score are significantly and negatively associated with parental absence. For girls, parental absence decreases the academic test score heavily, and the likelihood of student’s educational aspiration also significantly reduces.

Next, we examine the potential effect of parents’ education. The corresponding results are presented in Panel B of Table 7. The coefficients of parental absence for students with at least one better-educated parent are statistically insignificant (The educated parents are whose parents who have at least a college degree). The coefficients of parental absence are all much larger in magnitude and statistically significant for the students whose parents are not better-educated. Such results suggest that students whose parents are not educated suffer the adverse effects of separating from their parents. One possible explanation is that better-educated parents are able to earn more money by working outside, which increases an educational investment in children to offset the lack of parental absence.

Given the differences between urban and rural children in many aspects, we also examine the patterns of the effects on urban and rural students. As shown in Panel C of Table 7, parental absence has a larger negative effect on the outcomes of left behind children in urban areas than their rural counterparts. While previous studies mainly focused on rural left behind children, our results suggest that urban left behind children also suffer adverse effects and deserve more attention.

### 5.5. Mechanism

The baseline and IV estimations show that parental absence has a significant adverse impact on educational outcomes of left behind children whereas there is no evidence that exposure to more LBCs students in a class will negatively affect the outcomes of classmates in the class. In this section, we investigate the possible mechanisms underlying the internal effects of a parental absence.

The CEPS data cover rich information about students’ self-assessments on mental health status, behaviors, and campus life as well as the interaction between student-student and student-teacher, which enables us to conduct a series of tests to explore further the black box inside the effects of parent-child separation.

We first look at the effects of parental absence on students’ mental health. Some studies have shown that left-behind children have mental health problems [7,32,33,34,35]. To measure student’s mental health outcomes, we use students who represent five personnel items. The CEPS asked students about the frequency of the following feelings during the previous seven days on a scale from 1 to 5: Depressed, blue, unhappy, life is meaningless, and pessimistic. For regression, the dependent variables are dummy variables equal to 1 if the answers are “often” or “always,” 0 otherwise. Table 8 presents the estimated effects on students’ mental health. Though most of the R-square is less than 5%, all the coefficients are negative and statistically significant, which suggests that parental absence reduces the student’s mental health.

The CEPS also investigates students’ behaviors and campus life, which helps us explore the potential mechanisms of student’s effort in study, teacher-student interactions, student-student interactions, and classroom environment. As shown in Table 9, the CEPS asked students whether they agree with the following statement about campus life on a scale from 1 to 4. In regressions, the responses are dummy variables equal to 1 if the answers are “comparatively agree” or “strongly agree” and 0 otherwise. The effects are statistically significant. LBCs students are more likely to be late for school and less likely to receive praise from the head teacher. LBCs students may also have problems in communication with their classmates. Overall, we find supportive evidence that parental absence will reduce student’s effort, the frequency of teacher-student interactions and student-student interactions, and worsen the classroom environment.

We also use alternative aggregated variables as the measures for mental health and students’ perception of campus life to check the validity of the above mechanism analysis. For the mental health variable, we simply aggregate the response scores for five questions (i.e., depressed, blue, unhappy, life is meaningless, and pessimistic) to obtain a numerical variable that ranges from 5 to 25. The higher is the variable, the more severe is mental health. For the perception of campus life, we clarify two kinds of variables. We aggregate the response scores for three questions (i.e., late for school, feel bored, and hope to transfer school) to a numerical variable of negative feedback that ranges from 3 to 12. The higher the negative feedback variable is, the unhappier campus life is. We aggregate the response scores for six questions (i.e., “praises from headteacher,” “friendly classmates,” “easy to get along with,” “pleasant learning environment,” “activity participation,” and “feel close to students”) to a numerical variable of positive feedback that ranges from 6 to 24. The higher the positive feedback is, the happier campus life is. As shown in Table 10, we find that the findings using aggregate measures as dependent variables are basically consistent with those using separate measures as dependent variables listed in Table 8 and Table 9.

### 5.6. Long-Term Effects of Parental Absence: Alternative Data

In this sub-section, we focus on the long-term effects of parental absence on students’ educational development. Due to the limitations of the CEPS, we use alternative data from the 2010 and 2018 wave of the China Family Panel Studies (CFPS) (The CFPS is a national representative, longitudinal survey of Chinese communities, families, and individuals, launched in 2010 by the Institute of Social Science Survey (ISSS) of Peking University and followed-up every two years since, covering 25 provinces/municipalities/autonomous regions (excluding Hong Kong, Macao, Taiwan, Xinjiang, Tibet, Qinghai, Inner Mongolia, Ningxia, and Hainan)). We use a subsample of children who were in school in 2010 and aged 18+ in 2018. As before, parental absence is a dummy variable equal to 1 if the child was a left behind child in 2010. In the exercises, we further classify a father absence and a mother absence. Almost 13% of the children in our sample were left behind children in 2010. Among the left behind children, 66.2% were only absent of father’s care, 12.96% were only absent of mother’s care, and the remaining 20.83% were absent of parental care.

The measures for educational outcomes in this sub-section include two dummy variables denoting whether graduating from senior high school and whether entering college, respectively. The information on educational attainment is extracted from the adult data of CFPS 2018. To be consistent with the previous analysis, we also control the same set of individual characteristics, but we cannot control class characteristics due to data limitations.

Table 11 reports the OLS estimation results. Columns (1) and (3) test the long-term effects of parental absence on children’s educational development measured as senior high school graduation and college enrollment. The coefficients of parental absence are negative but statistically insignificant. As an attempt to understand the role of father and mother, we separately estimate the effects of fathers’ and mothers’ absence in Columns (2) and (4). The results suggest that the effect of a mother’s absence is more significant and harmful than that of a father’s absence on the outcomes of children. One possible reason is that a father may make more money than a mother to increase the educational investment, which may outweigh the effect of reduced time put into child learning. The other possible reason is that the role of the mother is to take care of the children in the Chinese traditional family, but the father is outside of fostering the children. Lastly, we explore the effect of the length of parental absence in Columns (3) and (6). Specifically, the length of a father’s/mother’s absence is defined as a child’s accumulated months of parental absence by the survey year (i.e., 2010). It shows that the length of a mother’s absence is negatively associated with children’s educational attainment, but there is no significant effect of the length of a father’s absence.

## 6. Conclusions

Using two large-scale survey datasets collected from China, this paper investigates the effects of parental absence on the educational development of left-behind children. Our IV estimation results show that a parental absence has significant negative impacts on the short-term and long-term educational outcomes of left behind children. Left behind children have a lower cognitive test score and academic test score, and they are also less likely to attend a college. Potential mechanism analyses show that a parental absence may result in a less healthy mental status of children and reduces children’s efforts in the class. In particular, a mother’s absence seems to have persistent negative effects on children development. This study also investigates and finds no significant evidence that the exposure to left-behind children in class lowers the in-school outcomes of children. This paper shows that a parental absence has negative effects on children’s education development, which may also affect China’s future human capital accumulation. One implication of the finding is that the governments should pay more attention to the LBCs and develop constructive policies to improve the situation of LBCs.

Although growing literature have devoted attention to the issue of parental absence, we contribute to this literature in several aspects in comparison with the previous studies. First, we look at the effects of parental absence on the rural left behind children as well as their urban counterparts whereas most previous studies focus on the left behind children in rural areas [7,14,15,16,18,34,35]. Second, we also examine the potential persistent effects of parental absence, while most previous studies only investigate the short-term effects [14,15,16,18]. Third, we further explored potential mechanisms underlying the effect. Besides mental health mechanism that is well documented in previous studies [7,11,32,33,34,35], we further explored other potential mechanisms such as in-school interactions. Lastly, we examine the external effect of left behind children in a class on the outcomes of their classmates even though the effect is found to be statistically insignificant.

This study also has limitations that can be improved in the future. First, the left behind children in the CEPS 2013–2014 are students in the compulsory education period, which indicates that we cannot look into the consequences of parent-child separation during early childhood. An early stage environment, especially before age five, seems to be a critical period of human development [1,36,37]. Second, the data lack some important family-level information (such as family income), which may also play a crucial role in parental migration decision.

## Figures and Tables

**Table 1:** Summary statistics of the variables.

Variables	Definitions	Observation	Mean	S.D.
Dependent variables				
Cognitive test score	Cognitive test score provided by CEPS	10,532	10.237	3.851
Average test score	Average test sore from mid-term examinations provided by each school	10,532	81.022	25.559
Chinese test score	Chinese test score provided by each school	10,532	82.861	21.494
Math test score	Math test score provided by each school	10,532	79.147	32.248
English test score	English test score provided by each school	10,532	81.057	30.336
Educational aspiration	Whether having the aspiration to at least attend college	10,532	0.812	0.391
Key independent variables				
Parental absence	Whether being an LBC (Yes = 1)	10,532	0.198	0.398
Share of LBCs	Share of LBCs in class	10,532	0.201	0.154
Individual and class variables				
Age	Student’s age	10,532	13.949	1.350
Ethnicity	Whether being a Han Chinese (Han = 1)	10,503	0.893	0.310
Only child in family	Whether being an only child (Yes = 1)	10,532	0.495	0.500
Gender	Student’s gender (Male = 1)	10,532	0.508	0.500
Hukou type	Whether having an urban Hukou (Rural = 1)	10,532	0.467	0.499
Mother with a college education	Whether mother with tertiary education (Yes = 1)	10,532	0.168	0.374
Father with a college education	Whether father with tertiary education (Yes = 1)	10,532	0.224	0.417
Class size	Number of students in class	10,532	47.277	13.277
Share of better-educated parents	Share of students whose parents both with tertiary education in class	10,532	0.143	0.174
Potential channels variables				
Depressed	Whether being depressed in the last week	10,289	2.233	0.991
Blue	Whether being blue in the last week	10,263	1.982	1.053
Unhappy	Whether being unhappy in the last week	10,277	2.280	1.041
Life is meaningless	Whether feeling life is meaningless in the last week	10,246	1.745	1.061
Pessimistic	Whether feeling pessimistic in the last week	10,273	2.026	1.029
Late for school	Always being late for school	10,452	1.242	0.612
Praises from headteacher	Class headteacher often praises me	10,396	2.387	0.892
Friendly classmates	Classmates are friendly to me	10,414	3.296	0.805
Easy to get along with	Feeling easy to get along with	10,411	3.196	0.842
Pleasant learning environment	Class learning environment is pleasant	10,395	3.192	0.867
Activity participation	Often participating in activities organized by the school or class	10,411	2.842	1.008
Feel close to students	Feeling close to students in the school	10,337	3.006	0.923
Feel bored	Feeling bored in the school	10,363	1.622	0.858
Hope to transfer school	Hoping to transfer to another school	10,422	1.472	0.856

Source: The data source is the China Education Panel Survey (CEPS) 2013–2014. Only randomly-assigned classes are kept in the sample.

**Table 2:** Summary statistics of the outcome variables.

Variables	Non-LBCs Students		LBCs Students		Differences
	Mean	S.D.	Mean	S.D.	
Cognitive test score	10.413	3.821	9.522	3.891	0.891 ***
Average test score	82.106	25.303	76.628	26.119	5.478 ***
Chinese test score	83.604	21.258	79.851	22.179	3.753 ***
Math test score	80.477	31.720	73.755	33.783	6.722 ***
English test score	82.236	30.098	76.277	30.830	5.959 ***
Educational aspiration	0.822	0.383	0.774	0.419	0.048 ***
Observation	8448		2084		

Note: The data source is the China Education Panel Survey (CEPS) 2013–2014. The cognitive test score denotes a student’s test score obtained in the standard cognitive tests conducted by the survey project. The average test score denotes the average test score of three core subjects (Chinese, math, and English) conducted by each school. Chinese test score, math test score, and English test score denote the test scores in Chinese, Math, and English, respectively. The last column reports the differences in the means of corresponding outcomes between non-LBCs students and LBCs students. *** *p* < 0.01.

**Table 3:** Tests for the random assignment rules.

Variables	OLS	School Fixed Effects	Grade-by-School Fixed Effects
	(1)	(2)	(3)
Age	0.081	0.094	0.101
	(0.528)	(0.141)	(0.165)
Ethnicity	−0.430 ***	0.024	0.051
	(0.153)	(0.036)	(0.041)
Only child in family	−0.892 ***	−0.095	−0.130
	(0.075)	(0.082)	(0.086)
Gender	0.005	−0.034	−0.016
	(0.032)	(0.088)	(0.070)
Hukou type	0.737 ***	0.182	0.224 *
	(0.085)	(0.122)	(0.125)
Mother with a college education	−0.432 ***	−0.068	0.015
	(0.055)	(0.055)	(0.053)
Father with a college education	−0.501 ***	−0.209 ***	−0.105
	(0.061)	(0.073)	(0.068)

Notes: The data source is China Education Panel Survey (CEPS) 2013–2014. Robust standard errors reported in parentheses are clustered at the class level. Estimate in each cell is obtained from a separate regression in which the dependent variable is the student’s predetermined variable, as listed above. An ordinary least squares (OLS) estimation without any fixed effects is applied for regressions in Column (1). Regressions in Column (2) include a set of grade and school fixed effects. Specifications in Column (3) include grade fixed effects, school fixed effects, and grade-by-school fixed effects. *** *p* < 0.01, * *p* < 0.1.

**Table 4:** Effects of parental absence on students’ academic outcomes.

Variables	Cognitive Test Score		Academic Test Score		Educational Aspiration	
	(1)	(2)	(3)	(4)	(5)	(6)
Parental absence	−0.188 **	−0.167 *	−3.033 ***	−2.612 ***	−0.029 ***	−0.022 **
	(0.095)	(0.093)	(0.514)	(0.493)	(0.010)	(0.010)
Share of LBCs	−0.231	0.043	−16.419	−12.137	−0.176	−0.149
	(1.517)	(1.477)	(10.328)	(7.946)	(0.110)	(0.095)
Control variables	No	Yes	No	Yes	No	Yes
Fixed effects	Yes	Yes	Yes	Yes	Yes	Yes
Observations	10,532	10,503	10,532	10,503	10,532	10,503
R-squared	0.320	0.333	0.524	0.561	0.111	0.136

Note: The data source is the China Education Panel Survey (CEPS) 2013–2014. Robust standard errors reported in parentheses are clustered at the class level. A full set of individual and class characteristics listed in Table 1, grade fixed effects, school fixed effects, and grade-by-school fixed effects are included in each regression. Share of LBCs indicates the share of left behind children in class. “Yes” indicates that we control these variables in estimation while “No” means we do not control these variables in estimation. *** *p* < 0.01, ** *p* < 0.05, * *p* < 0.1.

**Table 5:** IV Estimates of effects of parental absence on students’ academic outcomes.

Variables	Cognitive Test Score	Academic Test Score	Educational Aspiration
	(1)	(2)	(3)
Parental absence	−0.187 **	−2.669 ***	−0.024 **
	(0.093)	(0.491)	(0.010)
Share of LBCs	0.034	−12.163	−0.150
	(1.464)	(7.885)	(0.094)
F statistics of the first stage	206.44	206.44	206.44
Control variables	Yes	Yes	Yes
Fixed effects	Yes	Yes	Yes
Observations	10,503	10,503	10,503
R-squared	0.333	0.561	0.136

Note: The data source is the China Education Panel Survey (CEPS) 2013–2014. Robust standard errors reported in parentheses are clustered at the class level. A full set of individual and class characteristics listed in Table 1, grade fixed effects, school fixed effects, and grade-by-school fixed effects are included in each regression. “Yes” indicates that we control these variables in estimation. *** *p* < 0.01, ** *p* < 0.05.

**Table 6:** Effects of parental absence on test scores of different subjects.

Variables	Chinese	Math	English
	(1)	(2)	(3)
Panel A: OLS estimation			
Parental absence	−1.492 ***	−3.371 ***	−2.973 ***
	(0.353)	(0.723)	(0.583)
Share of LBCs	−10.159	−13.378	−12.874
	(6.300)	(10.695)	(8.751)
Pane B: IV estimation			
Parental absence	−1.409 ***	−3.499 ***	−3.099 ***
	(0.354)	(0.724)	(0.593)
Share of LBCs	−10.122	−13.437	−12.932
	(6.249)	(10.611)	(8.681)

Note: The data source is the China Education Panel Survey (CEPS) 2013–2014. Robust standard errors reported in parentheses are clustered at the class level. A full set of individual and class characteristics listed in Table 1, grade fixed effects, school fixed effects, and grade-by-school fixed effects are included in each regression. In panel A, we conduct an ordinary least squares (OLS) estimation; while in Panel B we use an instrumental variable (IV) estimation. *** *p* < 0.01.

**Table 7:** Heterogeneous effects of parental absence on student outcomes.

Variables	Cognitive Test Score	Academic Test Score	Educational Aspiration
	(1)	(2)	(3)
Panel A: by gender			
Boys	−0.238 *	−1.936 ***	0.007
	(0.130)	(0.691)	(0.014)
Girls	−0.184	−3.493 ***	−0.055 ***
	(0.125)	(0.680)	(0.015)
Panel B: by parents’ education			
Educated parents	0.105	−1.221	−0.014
	(0.269)	(1.336)	(0.020)
Parents without a college education	−0.213 **	−2.776 ***	−0.023 **
	(0.094)	(0.517)	(0.011)
Panel C: by Hukou type			
Rural students	−0.022	−1.813 ***	0.005
	(0.113)	(0.692)	(0.015)
Urban students	−0.393 ***	−3.467 ***	−0.050 ***
	(0.140)	(0.688)	(0.014)

Note: The data source is the China Education Panel Survey (CEPS) 2013–2014. Robust standard errors reported in parentheses are clustered at the class level. The estimate in each cell is obtained from a separate regression. A full set of individual and class characteristics listed in Table 1, grade fixed effects, school fixed effects, and grade-by-school fixed effects are included in each regression. The IV estimation is applied in columns (1)–(3). In Panel A, according to gender we divide the sample into male subsample and female subsample. In Panel B, according to parents’ education we divide the sample into educated parents subsample and less educated parents (without college education) subsample. In Panel C, according to Hukou type we divide the sample into rural subsample and urban subsample. Finally, we run separate regression for each subsample. *** *p* < 0.01, ** *p* < 0.05, * *p* < 0.1.

**Table 8:** The effect of parental absence on mental health.

Variables	Depressed	Blue	Unhappy	Life Is Meaningless	Pessimistic
	(1)	(2)	(3)	(4)	(5)
Parental absence	0.025 *	0.035 ***	0.047 ***	0.046 ***	0.040 ***
	(0.013)	(0.013)	(0.013)	(0.013)	(0.013)
Control variables	Yes	Yes	Yes	Yes	Yes
Fixed effects	Yes	Yes	Yes	Yes	Yes
Observations	10,503	10,503	10,503	10,503	10,503
R-squared	0.056	0.049	0.049	0.043	0.043

Note: The data source is the China Education Panel Survey (CEPS) 2013–2014. Robust standard errors reported in parentheses are clustered at the class level. The estimate in each cell is obtained from a separate regression. A full set of individual and class characteristics listed in Table 1, grade fixed effects, school fixed effects, and grade-by-school fixed effects are included in each regression. The IV estimation is applied in columns (1)–(5). “Yes” indicates that we control these variables in estimation. *** *p* < 0.01, * *p* < 0.1.

**Table 9:** The effect of parental absence on the perception of campus life.

Dependent Variables	Parental Absence	Observation
Late for school	0.022 ***	10,503
	(0.007)	
Praises from headteacher	−0.054 ***	10,503
	(0.013)	
Friendly classmates	−0.021 **	10,503
	(0.010)	
Easy to get along with	−0.018 *	10,503
	(0.011)	
Pleasant learning environment	−0.020 **	10,503
	(0.010)	
Activity participation	−0.038 ***	10,503
	(0.013)	
Feel close to students	−0.029 **	10,503
	(0.011)	
Feel bored	0.030 ***	10,503
	(0.011)	
Hope to transfer school	0.036 ***	10,503
	(0.054)	

Note: The data source is the China Education Panel Survey (CEPS) 2013–2014. Robust standard errors reported in parentheses are clustered at the class level. The estimate in each cell is obtained from a separate regression. A full set of individual and class characteristics listed in Table 1, grade fixed effects, school fixed effects, and grade-by-school fixed effects are included in each regression. Each cell in Column (1) represents a separate IV estimation in which the dependent variable is the students’ answers on the perception of campus life. *** *p* < 0.01, ** *p* < 0.05, * *p* < 0.1.

**Table 10:** Alternative measures for mental health and perception of campus life.

Variables	Mental Health	Positive Evaluation	Negative Evaluation
	(1)	(2)	(3)
Parental absence	0.575 ***	−0.307 ***	0.226 ***
	(0.119)	(0.100)	(0.050)
Control variables	Yes	Yes	Yes
Fixed effects	Yes	Yes	Yes
Observations	10,147	10,134	10,310
R-squared	0.074	0.150	0.129

Note: The data source is the China Education Panel Survey (CEPS) 2013–2014. Robust standard errors reported in parentheses are clustered at the class level. The estimate in each cell is obtained from a separate regression. A full set of individual and class characteristics listed in Table 1, grade fixed effects, school fixed effects, and grade-by-school fixed effects are included in each regression. The IV estimation is applied. “Yes” indicates that we control these variables in estimation. *** *p* < 0.01.

**Table 11:** Long-term effect of parental absence on children’s educational development.

Variables	Graduated from High School			Entered College		
	(1)	(2)	(3)	(4)	(5)	(6)
Parental absence	−0.041			−0.019		
	(0.033)			(0.034)		
Father’s absence		0.026			0.027	
		(0.036)			(0.038)	
Mother’s absence		−0.150 **			−0.122 **	
		(0.059)			(0.058)	
Length of father’s absence (log)			0.009			0.011
			(0.021)			(0.021)
Length of mother’s absence (log)			−0.067 **			−0.065 **
			(0.032)			(0.031)
Observations	1622	1622	1622	1622	1622	1622
R-squared	0.197	0.200	0.199	0.168	0.170	0.170

Note: The data sources are the 2010 and 2018 waves of the China Family Panel Studies (CFPS). Robust standard errors are reported in parentheses. The estimate in each cell is obtained from a separate regression. We also control variables such as age, ethnicity, only child in family, gender, Hukou type, mother with a college education, and father with a college education in each regression. ** *p* < 0.05.
